# Correlation Between Bioelectrical Impedance Analysis and Chest CT-Measured Erector Spinae Muscle Area: A Cross-Sectional Study

**DOI:** 10.3389/fendo.2022.923200

**Published:** 2022-07-19

**Authors:** Jie Cao, Didi Zuo, Tingting Han, Hongxia Liu, Wenwen Liu, Jia Zhang, Yurong Weng, Xian Jin, Zengai Chen, Yaomin Hu

**Affiliations:** ^1^ Department of Geriatrics, Renji Hospital, School of Medicine, Shanghai Jiaotong University, Shanghai, China; ^2^ Department of Radiology, Renji Hospital, Shanghai Jiao Tong University School of Medicine, Shanghai, China

**Keywords:** computed tomography, skeletal muscle mass, T12 erector spinae muscle area, L3 skeletal muscle mass area, bioelectrical impedance analysis

## Abstract

**Background:**

Skeletal muscle mass (SMM) plays an important part in diverse health and disease states. Bioelectrical impedance analysis (BIA) and computed tomography (CT) are available for its assessment. However, muscle mass assessed by BIA may be influenced by multiple factors. The erector spinae muscle area (ESA) on chest CT is recently presumed to be representative of SMM. This study aimed to derive BIA from the ESA and evaluate the magnitude of association (between ESA measured from chest CT) and BIA.

**Methods:**

Subjects hospitalized for health checkups between December 2020 and December 2021, having undergone both BIA (50 kHz, 0.8 mA) and chest CT, were included. ESA was quantified at the level of the 12th thoracic vertebra (T12-ESA) by a standardized semi-automated segmentation algorithm. Low SMM was defined using the Asian Working Group for Sarcopenia criteria. The association between T12-ESA and BIA was then evaluated. Stratified analyses by sex and BMI were also performed.

**Results:**

Among 606 included subjects (59.7 ± 16.6 years, 63.5% male), 110 (18.2%) had low SMM. BMI in low and normal SMM groups was 20.1 and 24.7 kg/m^2^, respectively. Current smoking, drinking, chronic obstructive pulmonary disease, and chronic renal dysfunction were more frequently seen in the low SMM group than in the normal SMM group. The final regression model included T12-ESA, weight, BMI, and age, and had an adjusted *R* ^2^ of 0.806 with BIA. In the validation group, the correlation between T12-ESA-derived BIA and BIA remained high (Pearson correlation = 0.899). Stratified analysis disclosed a stronger correlation between T12-ESA and BIA in male subjects than in female subjects (adjusted *R* ^2^ = 0.790 vs. adjusted *R* ^2^ = 0.711, *p* < 0.05), and a better correlation was observed in obese (BMI ≥ 30 kg/m^2^) compared with underweight (BMI < 18.5 kg/m^2^) subjects (adjusted *R* ^2 =^ 0.852 vs. adjusted *R* ^2^ = 0.723, *p* < 0.05). Additional analysis revealed a significant correlation between T12-ESA and skeletal muscle cross-sectional area at the 3rd lumbar vertebra (L3-CSA) (adjusted *R* ^2^ = 0.935, *p* < 0.001).

**Conclusions:**

CT-based assessment of ESA at the T12 level is feasible and correlated well with BIA, especially in male subjects and obese subjects.

## Introduction

Skeletal muscle, as the largest metabolic organ in the body, plays an indispensable role in hormone, metabolic health, and physical function. The reduction of skeletal muscle mass (SMM) along with the loss of muscle strength, a well-known condition called sarcopenia (1), increases the risk of disability, poor quality of life, and mortality (2). Therefore, an accurate noninvasive assessment of SMM is of particular concern for clinicians. However, there are currently no accepted objective measures of SMM.

To date, bioelectrical impedance analysis (BIA) is the preferred technique for SMM measurement in the 2019 Consensus Guidelines of the Asian Working Group for sarcopenia (AWGS), which has been applied in several studies (3, 4). The strengths of this approach include the following: it is inexpensive and easy to use (5), it is safe and noninvasive (6), it is portable, and it does not require highly trained operating personnel (7). However, the obvious drawbacks of BIA measurement (that is, susceptible to hydration status, recent activity, and time being horizontal (8)) limit its extensive use in clinical practice. Therefore, the search for more accurate methods without the above drawbacks should be a priority for sarcopenia research.

cRecently, computed tomography (CT) imaging with its excellent resolution of adipose tissue and skeletal muscles has shown a rapid increase in utilization in the evaluation of sarcopenia (9), as recommended by the Foundation for the National Institutes of Health Sarcopenia Project and multiple domestic and international guidelines (1, 10, 11). Through image analysis software, muscle and fat cross-sectional areas were determined according to the corresponding Hounsfield unit (HU) thresholds (12), which provided a reliable and objective measure of muscle mass. The third lumbar vertebra (L3) on abdominal CT is generally accepted as the standard level in the assessment of muscle mass because of its accuracy and robust correlation of the single slice measurement with whole-body volumes of muscle and adipose tissue (13, 14). However, for patients whose abnormalities are mainly restricted to the chest, the L3 level is not included on chest CT, and supplementary abdominal CT would result in both additional medical costs and radiation exposure. Thus, there is growing interest in the measurement of SMM at the level of the 12th thoracic vertebra (T12), which can be visualized not only on chest CT but also on abdominal CT. Of note, accumulating evidence indicates that the erector spinae muscle area at the T12 level (T12-ESA), used to identify thoracic low SMM, was also shown to be related to the muscle area at the L3 level (15, 16). More importantly, low SMM in the chest CT was demonstrated to be linked to poorer survival in various clinical diseases such as lung cancer (17), trauma (15), idiopathic pulmonary fibrosis (18), and many more (19). Nevertheless, the above-mentioned studies were more focused on the association between thoracic muscle and outcome, or the relationship between pectoralis muscle area and muscle mass derived from BIA. It remains unknown whether T12-ESA is in association with reduced SMM diagnosed by BIA.

We performed this cross-sectional study to establish the clinical association between T12-ESA and BIA, and further explore whether this association is stable across different sex and BMI ranges. Also, an additional analysis was conducted, including only patients who had both chest and abdominal CT, to assess the effect of using different diagnostic criteria based on the skeletal muscle cross-sectional area at the 3rd lumbar vertebra (L3-CSA).

## Methods

### Patients and Study Design

This retrospective, cross-sectional study was conducted in the inpatient unit of the Geriatrics Department of Renji Hospital for regular health checkups between December 2020 and December 2021. All adults (age > 18 years) who underwent BIA and chest CT were included in this analysis. The exclusion criteria were as follows: (1) age <18 years; (2) peripheral edema or taking diuretics within 1 month; (3) a history of surgery on the vertebrae or vertebral fractures; (4) neuromuscular diseases; and (5) oral, inhaled, or nasal glucocorticoid use. This study was approved by the Ethics Committee of Renji Hospital, School of Medicine, Shanghai Jiaotong University according to the Declaration of Helsinki, with a waiver of consent provided, given the retrospective observational design.

### BIA and Chest CT Scan

BIA (Euromedix, Leuven, Belgium) was performed in patients in the fasted state, after emptying their bladder, and after a rest of ≥15 min. We obtained limb BIA instead of trunk BIA by means of placing four electrodes on the limbs (two on the wrist and two on the ankle). BIA was normalized to the patient’s height squared (m^2^) and indexed in kg/m^2^.

Two multidetector CT scanners (Revolution 256 and Lightspeed 64; GE Healthcare) were used for all examinations. Scanning parameters were the same as the manufacturer’s standard recommended pre-setting for a thorax routine. Images were reconstructed with a 1-mm slice thickness in all cases with soft tissue kernel. The sum of cross-sectional areas of the erector spinae muscles at the T12 pedicle level of chest CT was used to generate T12-ESA. Assessment of cross-sectional areas of the muscle (including the psoas, erector spinae, quadratus lumborum, transversus abdominis, external and internal obliques, and rectus abdominis muscles) at the third lumbar vertebra (L3) pedicle level on abdominal CT was counted as L3-CSA. All images were analyzed by two trained observers (JC and ZC) in a semi-automated manner with the SliceOmatic V4.3 software (Tomovision, Montreal, Canada), which enables specific tissue demarcation using predefined CT HU thresholds: muscle, −29 to 150 HU (20); subcutaneous fat, −190 to −30 HU. Finally, muscle areas were computed for each tissue pixel by summing tissue pixels and multiplying the sum by the pixel surface area. The intra-class correlation coefficient (ICC) of the intra-observer agreement in T12-ESA was 0.972, and the ICC of the inter-observer reliability was 0.970. Muscle radiodensity estimation, also known as mean muscle radiation tissue attenuation (HU), was estimated for the entire cross-sectional area at L3, which correlates with the triglyceride content of muscle.

### Data Collection

Patient data were collected and maintained in an electronic database. For each patient, the following data were collected: age; sex; height; weight; BMI; smoking history; alcohol consumption; concomitant diseases including diabetes, hypertension, chronic obstructive pulmonary disease (COPD), and chronic renal failure (CRF); and blood test including hemoglobin, serum albumin, total cholesterol, triglycerides, serum creatinine, cystatin C, and glycated hemoglobin (HbA1c). Demographic data on BMI and height were documented within 24 h after admission. Current smoking was defined as having smoked at least 100 cigarettes during their lifetime and now smoking every day or some days. Current drinking was defined as self-reported some alcohol use of more than once per week.

### Statistical Analysis

The study population was randomly divided into low SMM and normal SMM groups according to the criteria of BIA set by the Asian Working Group for Sarcopenia (AWGS). Categorical variables are presented as numbers and percentages. Continuous variables are expressed as means ± SD for data with normal distribution and median (IQR) for non-normally distributed data. We compared means and proportions between groups by using Student’s test, analysis of variance, or the *χ* ^2^ test, as appropriate.

First, we split the entire sample into training (75% of the sample) and validation groups (25% of the sample). The training group was used to develop a formula to predict BIA derived from T12-ESA through stepwise multiple regression analysis; BIA was modeled as the dependent variable, with independent variables including T12-ESA, as well as BMI, sex, and age. Variable selection was based on combining automatic backwards and forwards stepwise multiple regression to obtain the model with the minimum Akaike information criterion. The validation group was used to test the performance of this formula, and the Pearson correlation coefficient was conducted to check the agreement between the observed BIA and BIA derived from T12-ESA. To assess the quality of this prediction model, we calculated the adjusted *R* ^2^ correlation. The range of *R* ^2^ correlation varies between 0 and 1, with the higher level showing higher correlation. An *R* ^2^ value < 0.3 is generally considered no or very weak correlation. An *R* ^2^ value range from 0.3 to 0.5 is generally considered a weak correlation, and an *R* ^2^ value > 0.7 is considered a strong correlation. In both validation and training groups, a Bland–Altman plot was additionally drawn to test the systematic bias across the range of BIA values, and further subgroup analyses by sex were performed.

Furthermore, we evaluated the accuracy of this formula across different subgroups stratified by BMI to differentiate low SMM patients using the AWGS criteria. Pearson correlation was employed for this evaluation. Finally, we ran an additional analysis, limited to patients who had concurrent chest and abdominal CT, to assess whether T12-ESA correlated well with L3-CSA using a manual stepwise model and the Pearson correlation coefficient.

A two-sided *p* < 0.05 was considered statistically significant. Analyses were performed using R (mgcv, blockrand, and boot packages, R version 3.3).

## Results

### Patients and Characteristics

**Figure 1:**
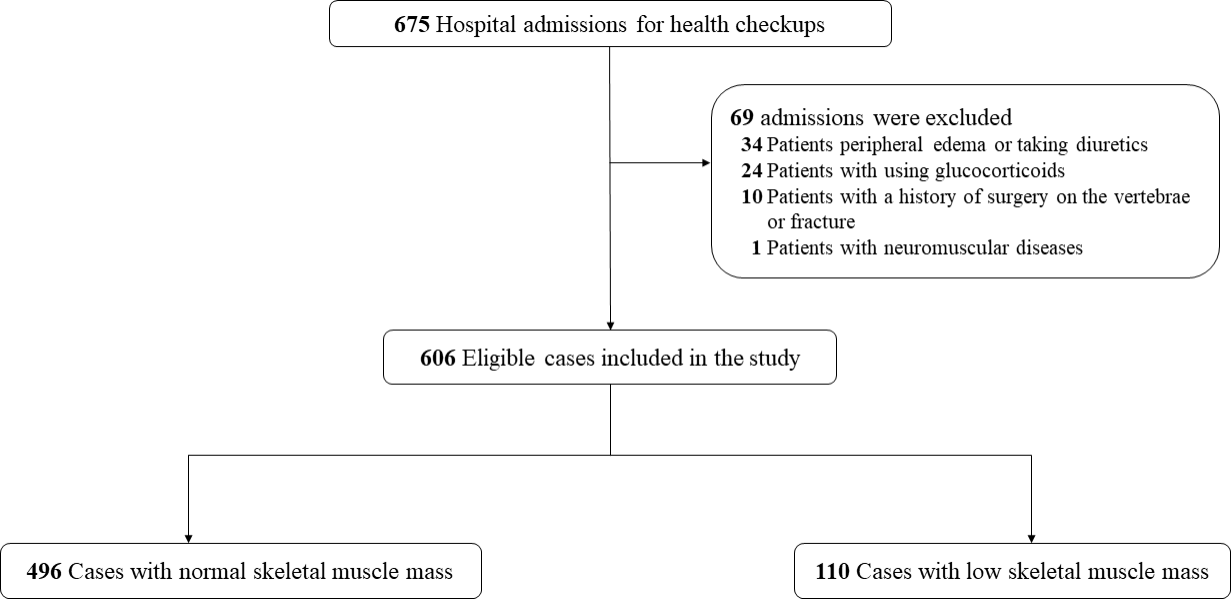
Flowchart of the study.

Of a total of 675 subjects admitted to the geriatric department during the study period, 69 patients met exclusion criteria, leaving 606 patients who were included in the study population (**Figure 1**). According to the cutoff values of BIA proposed by the Asian Working Group for Sarcopenia (AWGS), 110 (18.2%) patients and 496 (81.8%) patients were categorized as having low SMM and normal SMM, respectively. Baseline characteristics and the CT muscle parameters for both groups are compared in **Table 1**. As predicted, individuals with low SMM were older and had lower BMI, and current smoking and drinking were more frequent. No significant differences were observed in sex, and in the proportion of patients with diabetes and hypertension. However, we found that a higher percentage of patients had COPD and CRF among individuals with low SMM (both all *p* < 0.001). The level of hemoglobin, triglycerides, and albumin was significantly higher in the normal SMM group; the opposite was observed for cystatin C (*p* < 0.001). Of the CT muscle parameters, HU values in male subjects with low SMM were significantly lower than those with normal SMM, but there was no significant difference observed in female subjects. L3-CSA and T12-ESA were markedly lower in subjects with low SMM.

**Table 1:** Comparison of baseline characteristics and CT muscle parameters in the study population with low SMM and normal SMM .

Characteristics	All subjects *N* = 606	Normal SMM *N* = 496 (81.8%)	Low SMM *N* = 110 (18.2%)	*p*-value
BIA (kg/m^2^)	7.2 ± 1.1	7.5 ± 1.0	5.9 ± 0.8	<0.001
Male	7.8 ± 0.9	8.1 ± 0.6	6.3 ± 0.6	<0.001
Female	6.2 ± 0.7	6.4 ± 0.5	5.2 ± 0.4	<0.001
Age (years)	59.7 ± 16.6	57.2 ± 15.3	70.9 ± 17.6	<0.001
BMI (kg/m^2^)	23.8 ± 3.8	24.7 ± 3.4	20.1 ± 2.7	<0.001
Male, *n* (%)	378 (62.4)	315 (63.5)	63 (57.3)	0.222
**Comorbidities**				
Current smoking, *n* (%)	109 (19.0)	97 (20.8)	12 (11.2)	0.021
Current drinking, *n* (%)	72 (12.5)	67 (14.4)	5 (4.6)	0.006
Hypertension, *n* (%)	226 (38.9)	178 (37.6)	48 (44.4)	0.190
Diabetes mellitus, *n* (%)	144 (24.4)	110 (22.8)	34 (31.5)	0.152
Hyperlipidemia, *n* (%)	134 (24.4)	119 (26.9)	15 (14.0)	0.005
COPD, *n* (%)	20 (3.5)	11 (2.4)	9 (8.3)	0.002
CKD, *n* (%)	39 (6.8)	22 (4.8)	17 (15.7)	<0.001
**Laboratory tests**				
Hemoglobin (g/L)	132.5 ± 19.5	134.9 ± 18.9	121.4 ± 18.3	<0.001
Albumin (g/L)	41.2 ± 4.6	41.5 ± 3.8	40.1 ± 7.1	0.004
Triglycerides (mmol/L)	1.3 (0.9–2.0)	1.4 (1.0–2.1)	1.0 (0.7–1.4)	<0.001
Total cholesterol (mmol/L)	4.6 (3.9–5.3)	4.7 (3.9–5.3)	4.44 (3.7–5.2)	0.108
Serum creatinine (μmol/L)	64 (53–75)	64 (54–75)	63 (50–76)	0.693
Cystatin C (mg/L)	0.9 (0.8–1.1)	0.9 (0.8–1.0)	1.0 (0.9–1.4)	<0.001
HbA1c (%)	5.9 ± 1.0	5.9 ± 1.0	5.7 ± 1.0	0.166
**CT muscle parameters**				
HU	41.2 ± 7.6	42.1 ± 7.3	37.34 ± 7.8	<0.001
Male	42.4 ± 7.7	43.6 ± 6.8	36.53 ± 9.0	<0.001
Female	39.3 ± 7.1	39.5 ± 7.4	38.4 ± 5.7	0.346
T12-ESA (cm^2^)	32.9 ± 10.0	34.9 ± 9.6	23.8 ± 6.1	<0.001
Male	37.0 ± 9.9	39.31 ± 8.7	25.27 ± 6.7	<0.001
Female	26.0 ± 5.4	27.1 ± 5.1	21.9 ± 4.6	<0.001
L3-CSA (cm^2^)	114.9 ± 33.8	124.8 ± 35.0	95.6 ± 20.6	<0.001
Male	129.6 ± 33.6	141.3 ± 33.2	105.7 ± 18.8	<0.001
Female	91.0 ± 15.5	96.9 ± 13.9	80.4 ± 12.4	<0.001

Data are presented as median (interquartile range), n (%), or mean ± standard deviation.

BIA, bioelectrical impedance analysis; BMI, body mass index; COPD, chronic obstructive pulmonary disease; CKD, chronic kidney disease; CSA, cross-sectional area; ESA, erector spinae muscle area; HbA1c, glycated hemoglobin; HU, Hounsfield unit; SMM, skeletal muscle mass.

### Generate Equation to Predict BIA From T12-ESA

To derive BIA from T12-ESA, the subjects were divided into training and validation groups, which showed no significant differences with regard to age, sex, BMI, current smoking status, or CT muscle measurements (**Table S1**). The unadjusted *R* ^2^ correlation between BIA and T12-ESA was 0.66 in the training group (**Table 2**). The fully adjusted final equation model is presented as follows:


$$\text{BIA}=3.227+0.019*\text{T}12-\text{ESA}\left({\text{cm}}^{2}\right)+0.103*\text{BMI}\left(\text{kg}/{\text{m}}^{2}\right)+0.919*\left(\text{if male}\right)-0.006*\text{age}$$

**Table 2:** Stepwise model building in the training group to generate an equation to convert T12-ESA to BIA.

Independent variable	Adjusted *R* ^2^	Beta	*p*-value
**Model 1**	0.664		
T12-ESA*		0.001	<0.001
**Model 2**	0.731		
T12-ESA*		0.040	<0.001
BMI (kg/m^2^)		0.084	<0.001
**Model 3**	0.794		
T12-ESA*		0.022	<0.001
BMI (kg/m^2^)		0.113	<0.001
Sex (ref.: male)		0.794	<0.001
**Model 4**	0.806		
T12-ESA*		0.019	<0.001
BMI (kg/m^2^)		0.103	<0.001
Sex (ref.: male)		0.919	<0.001
Age (years)		−0.006	<0.001

*T12-ESA represents the erector spinae muscle area at the level of the 12th thoracic vertebra.

**Figure 2:**
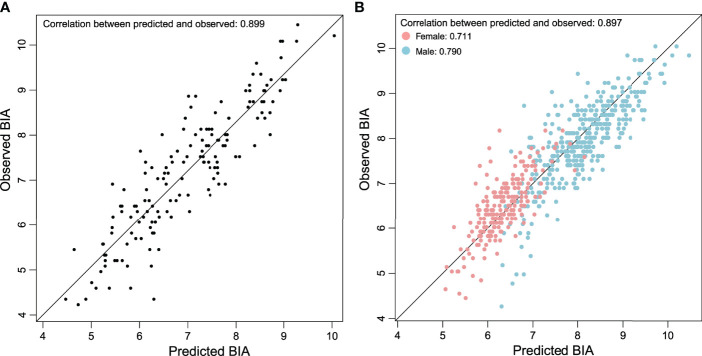
**(A)** Relationship between BIA derived from T12-ESA and observed BIA in the validation group composed of 152 cases. **(B)** Relationship between BIA derived from T12-ESA and observed BIA in all cases stratified by sex.

This equation included T12-ESA, BMI, sex, and age, and had an adjusted *R* ^2^ correlation of 0.806 among the BIA in the training group. Using the above equation, we then estimated BIA in the validation group. The correlation between T12-ESA and BIA was robust (*R* = 0.899, 95% CI 0.88–0.915, *p* < 0.001) (**Figure 2A**). As shown in **Figure 2B**, the correlation was strong between T12-ESA and BIA in all subjects (adjusted *R* ^2^ = 0.897; *p* < 0.0001). In the sex subgroup analysis, there was still a significant correlation between T12-ESA and BIA in male subjects (adjusted *R* ^2^ = 0.790, 95% CI 0.775–0.853, *p* < 0.0001) and female subjects (adjusted *R* ^2^ = 0.711, 95% CI 0.674–0.803, *p* < 0.0001) (**Figure 2B**). The correlation was stronger in male subjects than in female subjects (*p* < 0.05).

**Figure 3:**
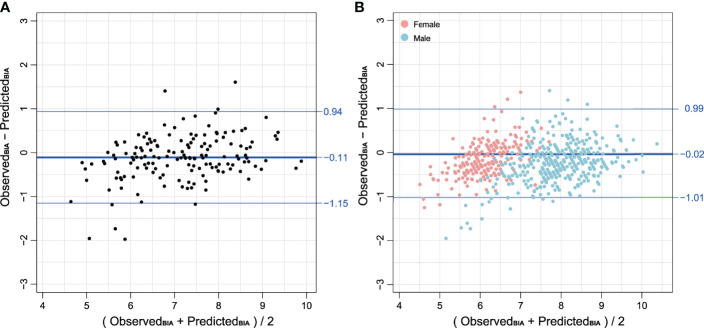
**(A)** Bland–Altman plot comparing BIA derived from T12-ESA and observed BIA in the validation group composed of 152 cases. **(B)** Bland–Altman plot comparing BIA derived from T12-ESA and observed BIA in all cases stratified by sex. Female cases are represented in red and male cases are represented in blue. Note: BIA units are kg/m^2^.

The Bland–Altman plots (**Figure 3A**) further supported the good agreement between T12-ESA and BIA across the range of values in the validation group, in which the mean difference was −0.11 kg/m^2^, and the limits of agreement related to the mean difference were −1.15 and +0.94 kg/m^2^. In male subjects and female subjects by subgroup, Bland–Altman plots demonstrated a mean difference ( ± standard deviation) of −0.01 ± 0.90 kg/m^2^ and −0.03 ± 1.03 kg/m^2^, respectively (**Figure 3B**).

### The Accuracy of the Prediction Equation in Detecting Low SMM

The above equation was then used to calculate predicted BIA in all cases. Using the AWGS criteria, the cases were divided into two groups: normal SMM and low SMM simultaneously stratified by BMI category. A better correlation was observed in obese (BMI ≥ 30 kg/m^2^) compared with underweight (BMI < 18.5 kg/m^2^) subjects (adjusted *R* ^2^ = 0.852 vs. adjusted *R* ^2^ = 0.723, *p* < 0.05) (**Table 3**). Next, we estimated the accuracy in distinguishing low SMM between predicted BIA derived from T12-ESA and observed BIA. In **Table 3**, we noted that no cases with a BMI greater than 30 kg/m^2^ were classified as low SMM, wherein there was complete agreement between predicted and observed BIA. In the overweight group (BMI 25–29.9 kg/m^2^) of predicted BIA, there were also no cases with low SMM versus 6 (3.5%) cases compared to observed BIA. Furthermore, in the low-weight group, this equation tended to a slight overestimation of low SMM (78.4% vs. 75.7%); however, an opposite effect was observed in the normal-weight group (15.7% vs. 20.9%) with an accuracy of 75.6% and 82.6%, respectively.

**Table 3:** The accuracy of predicted BIA derived from T12-ESA and observed BIA stratified by BMI category in all cases.

BMI category*	Predicted BIA		Observed BIA		Correlation
	Normal SMM	Low SMM	Normal SMM	Low SMM	
Total	520 (85.8%)	86 (14.2%)	496 (81.8%)	110 (18.2%)	0.897
<18.5 kg/m^2^	8 (21.6%)	29 (78.4%)	9 (24.3%)	28 (75.7%)	0.723
18.5–24.9 kg/m^2^	306 (84.3%)	57 (15.7%)	287 (79.1%)	76 (20.9%)	0.821
25–29.9 kg/m^2^	172 (100%)	0 (0%)	166 (96.5%)	6 (3.5%)	0.815
≥30 kg/m^2^	34 (100%)	0 (0%)	34 (100%)	0 (0%)	0.852

BMI, body mass index; BIA, bioelectrical impedance analysis; SMM, skeletal muscle mass.

*BMI category: underweight, <18.5; normal weight, 18.5–24.9; overweight, 25–29.9; and obese, ≥30.

### Relationship Between T12-ESA and L3-CSA

In an exploratory analysis aiming to assess the relationship between T12-ESA and L3-CSA, we limited the population to having concurrent chest and abdominal CT (*n* = 118). In multivariable linear regression analysis that included age, sex, BMI, and T12-ESA, each variable maintained an independent association with L3-CSA (*p* < 0.001; **Table S2**). The equations for the final model 4 are summarized as follows:


$$\text{L}3-\text{CSA}=35.075+1.729*\text{T}12-\text{ESA}\left({\text{cm}}^{2}\right)+2.117*\text{BMI}\left(\text{kg}/{\text{m}}^{2}\right)+25.217*\left(\text{if male}\right)-0.503*\text{age}$$

This model better predicted L3-CSA than individual T12-ESA and model 3 consisting of BMI, sex, and T12-ESA (*R* ^2^ = 0.869 vs. *R* ^2^ = 0.738 and *R* ^2^ = 0.824, respectively; **Table S2**). **Figure S2** demonstrated good Pearson correlation coefficients of 0.935 between the predicted and observed L3-CSA. Bland–Altman analysis showed the mean difference (0) and 95% limits of agreement (−24.01, 24.01), confirming the high agreement between the predicted and observed L3-CSA (**Figure S3**).

### Correlation Between BIA Derived From T12-ESA and Observed BIA in the Different Age Groups

Finally, we performed a comparison of baseline characteristics and CT muscle measurements between younger (<65 years) and older (≥65 years) groups in **Table S3**. The adjusted *R* ^2^ correlation in the younger group and the older group was 0.824 and 0.790, respectively (**Figure S4**).

## Discussion

SMM contributes significantly to physical and metabolic health. Its size can be utilized as a biomarker reflecting the severity of disease or predicting prognosis. BIA, an approved method for skeletal muscle measurement, is recommended by different guidelines (1, 10, 20). Recently, CT, having the advantage of excellent resolution of skeletal muscles and adipose tissue, has been proven to be accurate in assessing SMM in various diseases (21–23). To our knowledge, this is the first report of the association between T12-ESA and BIA.

In this study, we generated a formula to derive BIA using the ESA obtained from chest CT and demonstrated a good correlation between the BIA and T12-ESA-based assessment of BIA in adjusted models including sex, BMI, and age. Not surprisingly, adding these variables improved the performance of the model, given their association with body composition.

In our study, the prevalence rate of low SMM in hospitalized people was 18.2%. Prevalence estimates for low SMM vary widely in different clinical settings and diverse population characteristics. Therefore, our prevalence data are not directly comparable with other studies. However, low SMM becomes progressively more common among older subjects. In a European study, the prevalence of low SMM can be as high as 29% in older community-dwelling populations (24). Another prospective observational study in China showed that the rate was 17.0% in elderly patients (25). Consistent with findings from other studies (26–29), low SMM patients were older, had lower BMI, and included a higher proportion of smoking, drinking, COPD, and CRF. Cystatin C is a member of the cystatin superfamily and is produced by all nucleated cells. Patients with chronic interstitial nephropathy showed lower serum levels of cystatin C values defined as cystatin C/creatinine rate. Recent studies showed that the serum creatinine-to-cystatin C ratio predicts SMM and strength. However, the specific mechanisms of this relation are not yet clear. A critical finding of our study is that measures of muscle mass assessed by T12-ESA and BIA correlated more strongly in male subjects compared with female subjects. Only a few studies have assessed the gender-related differences in measuring BIA. In a large sample of the general UK population, BIA showed more overestimation of SMM in female subjects than in male subjects (2.5% vs. 1.9%) (30). In addition, another recent paper aiming to identify low skeletal muscle surface areas gave a similar profile; that is, BIA correlated better with CT scan in men than in women (31). However, further larger and dedicated studies are required to confirm our findings and elucidate underlying mechanisms.

Currently, several modalities are available for the estimation and quantification of muscle mass. However, these frequently utilized methods have limitations and drawbacks. First, anthropometry, as an indirect quantification of muscle mass, is particularly susceptible to individual and obesity-related prediction errors and is prone to overestimate muscle mass (32). Second, dual-energy x-ray absorptiometry (DEXA), which EWGSOP-2 recommends as the gold standard for quantification of muscle mass, is insensitive to small changes compared to CT and is unable to measure intramuscular adipose tissue (3, 33). Third, BIA, despite the lack of standardized methods for estimation and being vulnerable to hydration status and multiple diseases, is often used as a portable alternative to DEXA. Fourth, cross-sectional imaging, such as CT, could precisely quantify total and fat-free SMM (14). However, discretionary use of CT could confer additional radiation exposure and extra cost. Taken together, to date, no available single technique could fulfill all the requirements for the measurement of muscle mass. Each has its own merits and shortcomings. Moreover, all the above modalities are not fully standardized. Thus, a gold standard method for the measurement of muscle mass has not been established yet.

Interestingly, we found that BIA in subjects with high BMI showed a better correlation with T12-ESA than in low-BMI subjects. This finding differs from those of previous studies that reported that BIA is less accurate at high BMI levels in assessing body composition (4, 8, 34). The possible reason is the unavailability of BIA in obese or underweight subjects and the inhomogeneity of body compartments. Of note, though, because of different comparators (DEXA in previous ones; T12-ESA in ours), a direct comparison between studies is difficult. Yet, ESA as a major muscle is instrumental in respiration and maintenance of an erect posture. Although a study has previously shown that T12-ESA is affected by age (35), muscle measurements using T12-ESA have the advantage that its size depends more on the physical activities, not on simple gross body weight (36), and can be easily measured for quantification on chest CT scan. Thus, it is plausible that T12-ESA might outperform BIA regarding quantification of SMM parameters specifically in subjects with extremes of body mass. Also, perhaps this could alternatively explain why T12-ESA and L3-CSA reached an excellent correlation based on the CT scan. However, these results need to be further validated in larger populations.

There are several limitations associated with the current investigation that must be addressed. First, we did not compare BIA to all skeletal muscles at the level of T12, which is also used as a measure of muscle mass. However, the ESA was selected because it is easily measurable (22) and is required for locomotion (37) and previous studies have demonstrated its application (9, 38, 39). Second, data were collected retrospectively, with the variables regarding the muscle strength or physical performance being unavailable. Thus, we cannot categorize subjects as sarcopenic and propose a cutoff value for sarcopenia in this population. Third, we generated the prediction formula from people who underwent checkups, which accounted for a great heterogeneity in the characteristics including a large age range. Nonetheless, this heterogeneity represents real clinical settings. Fourth, this was a single-center study. There has been no external validation performed yet for this equation.

The assessment of muscle mass parameters’ CT based on a semi-automated method is a feasible alternative to BIA. A good correlation of BIA and T12-ESA was found in male subjects and obese subjects. T12-ESA demonstrated an excellent relationship with L3-CSA, which could serve as a promising imaging biomarker for the assessment of skeletal muscle on chest CT scans.

## Data Availability Statement

The raw data supporting the conclusions of this article will be made available by the corresponding authors, upon reasonable request.

## Ethics Statement

The studies involving human participants were reviewed and approved by Renji Hospital, School of Medicine, Shanghai Jiaotong University. Written informed consent for participation was not required for this study in accordance with the national legislation and the institutional requirements.

## Author Contributions

ZC, YH, and JC contributed to conception and design of the study. DZ and TH organized the data. TH, HL, and WL performed the statistical analysis. JC, DZ, and TH wrote the first draft of the manuscript. HL, WL, JZ, XJ, and YW wrote sections of the manuscript. All authors contributed to manuscript revision, read, and approved the submitted version.

## Acknowledgments

We thank Dr. Nicholas A. Tritos, from Neuroendocrine Unit, Massachusetts General Hospital, Harvard Medical School, USA, for his professional review of and suggestions to this manuscript.

## Funding

This work was supported by the National Nature Science Foundation of China (Grant 81870554) and Foundation from Renji Hospital, School of Medicine, Shanghai Jiaotong University (2019NYLYCP0102).

## Conflict of Interest

The authors declare that the research was conducted in the absence of any commercial or financial relationships that could be construed as a potential conflict of interest.

## Publisher’s Note

All claims expressed in this article are solely those of the authors and do not necessarily represent those of their affiliated organizations, or those of the publisher, the editors and the reviewers. Any product that may be evaluated in this article, or claim that may be made by its manufacturer, is not guaranteed or endorsed by the publisher.
