# Supplementary material for: Correlation Between Bioelectrical Impedance Analysis and Chest CT-Measured Erector Spinae Muscle Area: A Cross-Sectional Study
Source: Front Endocrinol (Lausanne). 2022 Jul 19;13:923200. doi: 10.3389/fendo.2022.923200 (PMC9343984; doi:10.3389/fendo.2022.923200)
Supplement: Supplementary file 1 [file DataSheet_1.docx]

Supplementary Material

# Supplementary Figures

#
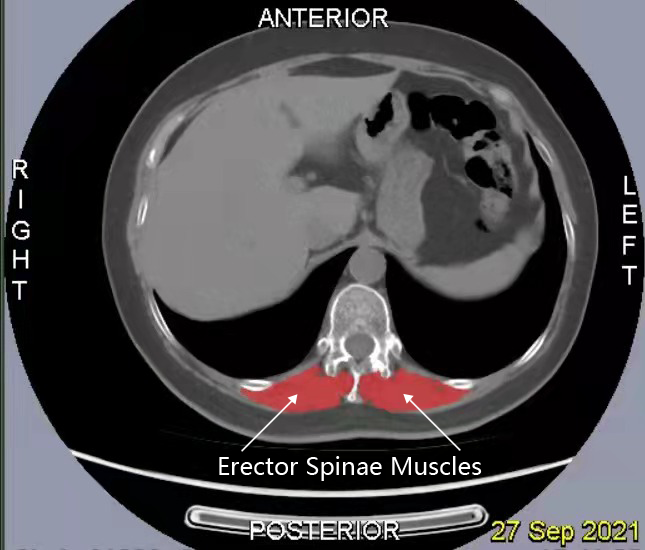


# Supplementary Figure 1 Computed tomography axial slice showing the segmentation of the erector spinae muscles at the level of the 12th thoracic vertebra. The areas of the muscles were aggregated to calculate the erector spinae muscle area (ESA).

#
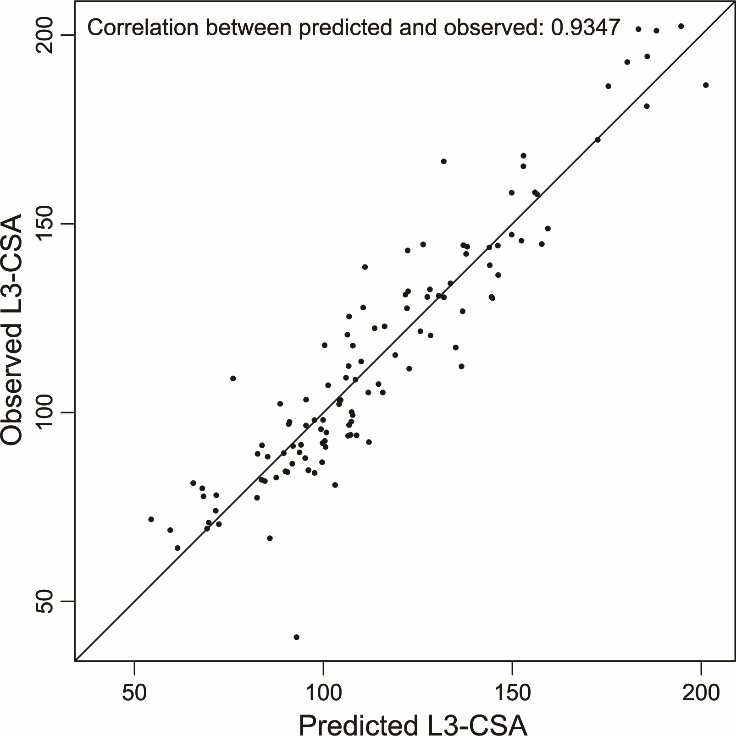


# Supplementary Figure 2 Relationship between L3-CSA and T12-ESA in a subset of 118 cases.

#
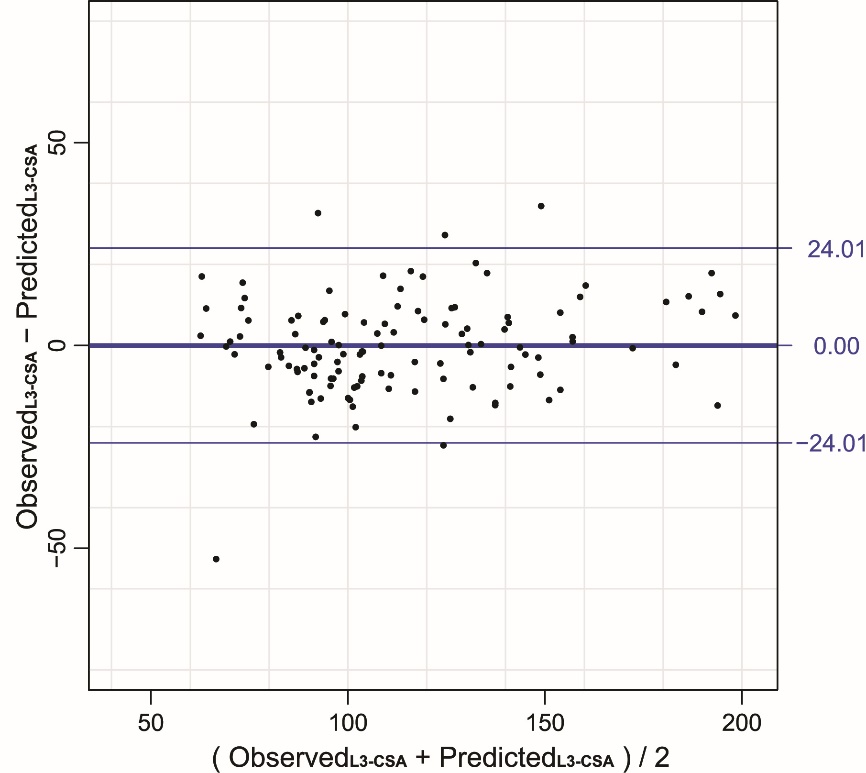


# Supplementary Figure 3 Bland-Altman plot comparing L3-CSA and T12-ESA in a subset of 118 cases.


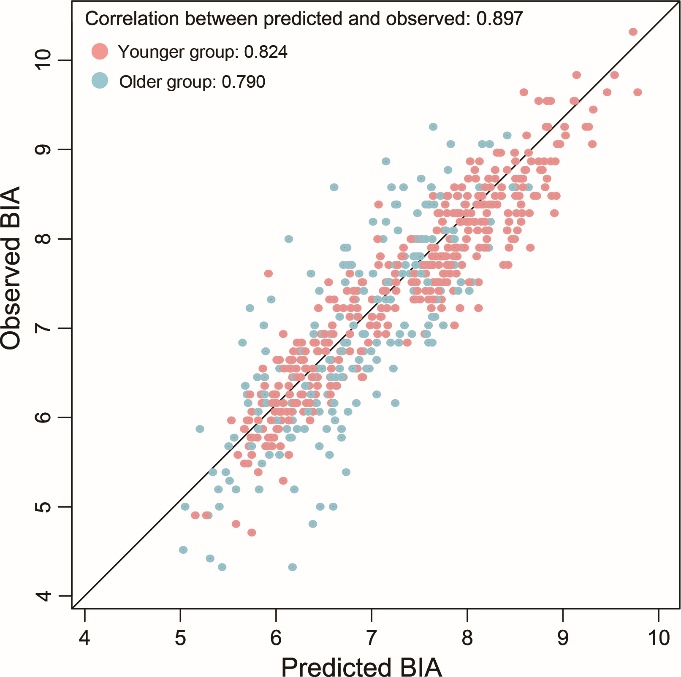


**Supplementary Figure 4** Correlation between BIA derived from T12-ESA and observed BIA in all cases stratified by younger group (younger than 65 years) and older group (aged 65 years and older).

# Supplementary Tables

# Supplementary Table 1 Comparison of baseline characteristics and CT muscle measurements between the training and validation groups.

| Characteristics | Training group  N = 450 | | Validation group  N = 156 | p-value |
| --- | --- | --- | --- | --- |
| BIA (kg/m^2^) | | 7.2 ± 1.1 | 7.1 ± 1.2 | 0.611 |
| **Demographic** | |  |  |  |
| Height (m) | | 1.67 ± 0.08 | 1.67 ± 0.07 | 0.790 |
| Age (years) | | 59.4 ± 16.0 | 60.6 ± 18.1 | 0.451 |
| BMI (kg/m^2^) | | 23.8 ± 3.6 | 23. 8 ± 4.2 | 0.874 |
| Male, n (%) | | 277 (61.6) | 101 (64.7) | 0.479 |
| **Comorbidities** | |  |  |  |
| Current smoking, n (%) | | 82 (19.3) | 27 (18.0) | 0.728 |
| Current drinking, n (%) | | 53 (12.5) | 19 (12.7) | 0.950 |
| Hypertension, n (%) | | 163 (38.0) | 63 (41.5) | 0.453 |
| Diabetes mellitus, n (%) | | 106 (24.2) | 38 (25.0) | 0.230 |
| Hyperlipidemia, n (%) | | 94 (23.3) | 40 (27.4) | 0.326 |
| COPD, n (%) | | 12 (2.8) | 8 (5.4) | 0.149 |
| CKD, n (%) | | 23 (5.5) | 16 (10.8) | 0.027 |
| **Laboratory tests** | |  |  |  |
| Hemoglobin (g/L) | | 133.8 ± 18.1 | 128.7 ± 22.7 | 0.041 |
| Albumin (g/L) | | 41.4 ± 4.8 | 40.7 ± 4.0 | 0.099 |
| Triglycerides (mmol/L) | | 1.3 (0.9-2.0) | 1.3 (0.9-2.0) | 0.682 |
| Total cholesterol (mmol/L) | | 4.7 (4.0-5.4) | 4.5 (3.8-5.2) | 0.126 |
| Serum creatinine (umol/L) | | 64 (53-74) | 66 (52-80) | 0.150 |
| Cystatin C (mg/L) | | 0.93 (0.82-1.06) | 0.93 (0.84-1.08) | 0.567 |
| HbA1c (%) | | 5.9 ± 1.0 | 5.8 ± 1.0 | 0.637 |
| **CT muscle parameters** | |  |  |  |
| HU | | 41.1 ± 7.5 | 41.6 ± 7.8 | 0.420 |
| T12-ESA (cm^2^) | | 32.8 ± 9.9 | 33.0 ± 10.4 | 0.872 |
| L3-CSA (cm^2^) | | 117.9 ± 32.2 | 107.1 ± 36.9 | 0.121 |

**Notes:** Data are presented as median (interquartile range), n (%), or mean ± standard deviation.

**Abbreviations:** BIA, bioelectrical impedance analysis; BMI, body mass index; COPD, chronic obstructive pulmonary disease; CKD, chronic kidney disease; CSA, cross-sectional area; ESA, erector spinae muscle area HbA1c, glycated hemoglobin; HU, Hounsfield unit; SMM, skeletal muscle mass

# Supplementary Table 2 Manual stepwise model building on a subset of 118 cases to generate an equation to convert T12-ESA to L3-CSA.

| Independent variable | Adjusted R^2^ | Beta | p-value |
| --- | --- | --- | --- |
| **Model 1** | 0.738 |  |  |
| T12-ESA* |  | 3.141 | <0.001 |
| **Model 2** | 0.775 |  |  |
| T12-ESA* |  | 2.685 | <0.001 |
| BMI (kg/m^2^) |  | 2.087 | <0.001 |
| **Model 3** | 0.824 |  |  |
| T12-ESA* |  | 2.430 | <0.001 |
| BMI (kg/m^2^) |  | 1.712 | <0.001 |
| Sex (ref, : male) |  | 16.889 | <0.001 |
| **Model 4** | 0.869 |  |  |
| T12-ESA* |  | 1.729 | <0.001 |
| BMI (kg/m^2^) |  | 2.117 | <0.001 |
| Sex (ref, : male) |  | 25.217 | <0.001 |
| Age (year) |  | -0.503 | <0.001 |

*T12-ESA represents the erector spinae muscle area at the level of the 12th thoracic vertebra.

**Supplementary Table 3** Comparison of baseline characteristics and CT muscle measurements between younger and older groups.

| Characteristics | Younger group*  N = 401 | | Older group*  N =205 | p-value |
| --- | --- | --- | --- | --- |
| BIA (kg/m^2^) | | 7.3 ± 1.1 | 6.8 ± 1.1 | <0.001 |
| **Demographic** | |  |  |  |
| Height (m) | | 1.68 ± 0.08 | 1.65 ± 0.08 | <0.001 |
| BMI (kg/m^2^) | | 24.3 ± 3.9 | 22.9 ± 3.4 | <0.001 |
| Male, n (%) | | 245 (61.1) | 133 (64.9) | 0.363 |
| **Comorbidities** | |  |  |  |
| Current smoking, n (%) | | 85 (22.5) | 24 (12.2) | 0.003 |
| Current drinking, n (%) | | 58 (15.3) | 14 (7.1) | 0.005 |
| Hypertension, n (%) | | 99 (26.0) | 127 (63.5) | <0.001 |
| Diabetes mellitus, n (%) | | 61 (15.6) | 83 (41.5) | <0.001 |
| Hyperlipidemia, n (%) | | 99 (27.3) | 35 (18.7) | 0.026 |
| COPD, n (%) | | 1 (0.3) | 19 (9.7) | <0.001 |
| CKD, n (%) | | 1 (0.3) | 38 (19.4) | <0.001 |
| **Laboratory tests** | |  |  |  |
| Hemoglobin (g/L) | | 136.8 ± 18.3 | 123.8 ± 18.9 | <0.001 |
| Albumin (g/L) | | 42.2 ± 3.2 | 39.3 ± 6.1 | <0.001 |
| Triglycerides (mmol/L) | | 1.4 (0.9-2.2) | 1.2 (0.8-1.7) | <0.001 |
| Total cholesterol (mmol/L) | | 4.8 (4.2-5.4) | 4.2 (3.5-5.0) | <0.001 |
| Serum creatinine (umol/L) | | 62.0 (52.0-72.0) | 68.5 (57.0-88.0) | <0.001 |
| Cystatin C (mg/L) | | 0.9 (0.8-1.0) | 1.1 (1.0-1.5) | <0.001 |
| HbA1c (%) | | 5.8 ± 0.9 | 6.1 ± 1.1 | <0.001 |
| **CT muscle parameters** | |  |  |  |
| HU | | 44.2 ± 5.6 | 35.5 ± 7.7 | <0.001 |
| T12-ESA (cm^2^) | | 35.5 ± 9.8 | 27.8 ± 8.2 | <0.001 |
| L3-CSA (cm^2^) | | 130.6 ± 37.7 | 102.9 ± 24.7 | <0.001 |

**Notes:** Data are presented as median (interquartile range), n (%), or mean ± standard deviation.

**Abbreviations:** BIA, bioelectrical impedance analysis; BMI, body mass index; COPD, chronic obstructive pulmonary disease; CKD, chronic kidney disease; CSA, cross-sectional area; ESA, erector spinae muscle area HbA1c, glycated hemoglobin; HU, Hounsfield unit; SMM, skeletal muscle mass

*All the subjects were divided into two groups: younger group (aged younger than 65 years) and older group (aged 65 years and older).
